# Uveitis associated with juvenile arthritis: a continued cohort study 40 years after uveitis onset

**DOI:** 10.1186/s12969-022-00704-8

**Published:** 2022-07-08

**Authors:** Angelika Skarin, Elisabet Berthold, Ola Rauer, Elisabeth Bengtsson-Stigmar

**Affiliations:** 1 Department of Ophthalmology, Clinical Sciences Lund, Skåne University Hospital, Kioskgatan 1, 22242 Lund, Sweden; 2 Department of Rheumatology, Clinical Sciences Lund, Skåne University Hospital, Kioskgatan 5, 222 42 Lund, Sweden

**Keywords:** Uveitis, Juvenile idiopathic arthritis, JIA, Juvenile chronic arthritis, JCA, Follow-up, Cohort, Mortality, Visual outcome

## Abstract

**Background:**

A third follow-up study, mean 40.7 years after uveitis onset, of a cohort originally consisting of 55 Swedish patients with uveitis associated with juvenile arthritis.

**Method:**

A retrospective study of the patients’ ophthalmic medical records. The results were compared to those of the same cohort previously studied at mean 7.2 and 24.0 years after uveitis onset. In the present follow-up study, 30 of the original 55 patients consented to participate. Of these, 26 had ophthalmic medical records that were reviewed.

**Results:**

In the 30 participants, active uveitis was seen in 43.4%, cataracts in 66.6% and glaucoma in 40.0%. When comparing data from previous follow-ups of the same cohort, a total of 61.8% were reported to have had cataracts at any of the three follow-ups, 29.0% had glaucoma or ocular hypertension and 12.7% had severe visual impairment in both eyes. At mean 40.7 years after uveitis onset 20% of patients in the original uveitis cohort were deceased. In 4 of the 11 deceased individuals, rheumatic disease was stated as the main cause of death, and in 3 it was considered a contributory factor in the patients deaths.

**Conclusions:**

Uveitis associated with juvenile arthritis can be active into midlife and possibly longer. Ocular complications and visual loss increased up to 40 years after uveitis diagnosis. The mortality rate of this cohort was higher than that of a corresponding Swedish population. Lifelong ophthalmic check-ups are probably necessary for patients diagnosed with this type of uveitis.

## Introduction

Juvenile idiopathic arthritis (JIA) is a rheumatic disease affecting 10–15 per 100,000 children per year in northern Europe [1–4]. Approximately 10–20% of JIA patients develop uveitis, which can be chronic and can cause blindness if insufficiently treated [5–8]. A low age at disease onset and the presence of anti-nuclear antibodies (ANA) are associated with an increased risk for uveitis [5, 6].

Regular eye screening for the early detection of uveitis in all children diagnosed with JIA is a well-established practice in Sweden and other countries [9].

A limited number of reports have described the long-term prevalence of disease activity and ocular complications associated with this type of uveitis [5, 7, 10–13]. These studies have indicated that uveitis can still be active in a significant number of patients in early adulthood.

In our previous study the eye status of this same cohort consisting of 55 patients with juvenile arthritis-associated uveitis was reported at mean 7.2 and 24.0 years after uveitis onset. Main findings were that 24 years after onset 49% of the patients were reported to have active uveitis, and an increase in the prevalence of cataract and glaucoma was seen between 7 and 24 years [7].

The aim of the present study was to assess uveitis activity and the prevalence of ocular complications 40 years after uveitis onset in the previously studied cohort.

## Materials and methods

### Study design and ethical approval

This was a retrospective study based on data from ophthalmic medical records registered 2016–2017. The study was performed in accordance with the Helsinki Declaration. It was approved by the Regional Ethical Review Board for southern Sweden (2017/263) and the National Ethical Review Agency (2020–02134).

### The original cohort

The original cohort included all 55 patients (15.7%) who had uveitis of 350 patients with a Juvenile Chronic Arthritis diagnosis (JCA) registered at the Paediatric Rheumatology section, Lund University Hospital in Sweden between 1973 and 1982. The European League Against Rheumatism (EULAR) criteria were then used to classify the joint disease [1, 7]. The patients in this uveitis cohort were born between 1944 and 1981.

**Fig. 1:**
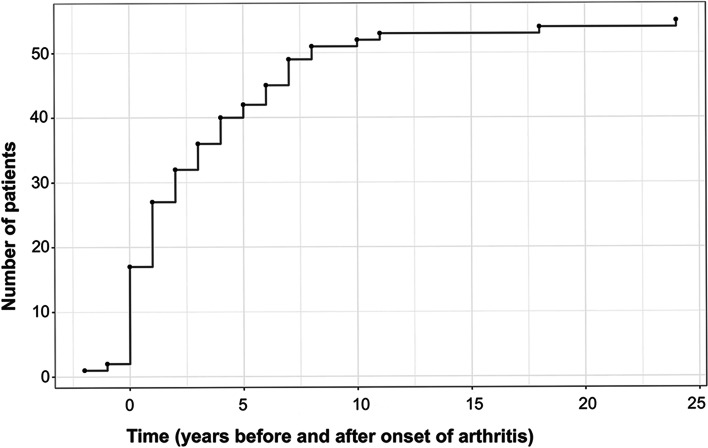
Onset of uveitis in years in relation to onset of arthritis in all 55 patients in the original cohort. On the horizonal x-axis “0” indicates the year of arthritis diagnosis. The vertical y-axis represents the number of patients in this cohort with uveitis onset each year. In two cases uveitis was diagnosed before arthritis. In fours case uveitis onset was from 10 to 24 years after arthritis. The joint diseases in three of these was classified as oligoarthritis and one as polyarthritis

Figure 1 illustrates the onset of uveitis, in years, in relation to the onset of JCA in the cohort.

Some of these data have been previously published [7].

Of the original 55 patients, 25 did not participate in the present follow-up study. Of these missing cases 11 had died before the present study commenced, another 10 did not respond to the invitation to participate in the study, and a further four were not registered as living in Sweden and their postal addresses could not be found.

### The present study

In the present study 30 patients consented to participate. Of these, 26 reported having ophthalmic medical records covering the years 2016–2017 and gave permission for these to be reviewed. Copies of all relevant ophthalmic records where then requested and recovered from clinics all over Sweden. The remaining four patients reported that they did not see an ophthalmologist regularly and had no records covering the last two years.

### Participating patients

At the time of this study the mean age of the 30 participating patients was 46.9 ± 6.9 years (median 45.5 years, range 38–72 years). The mean duration of joint disease was 42.9 ± 6.6 years (median 42.0 years, range 36–69 years) and the mean time from onset of uveitis was mean 40.7 ± 6.3 years (median 39.0 years, range 35–63 years).

### Definitions of active uveitis, cataracts, and glaucoma or ocular hypertension (G/OH)

A patient was considered to have active uveitis if described to have intraocular inflammation in need of topical corticosteroid treatment, or if the patient was described as having ongoing uveitis treatment with topical corticosteroid drops at any time during 2016 to 2017 at the discretion of the treating ophthalmologist.

Cataracts were considered present if clearly described in the patient records or in the previous follow-up studies for that specific individual [7]. The presence of an intraocular lens or aphakia in one or both eyes was interpreted as a consequence of previous cataracts and thus registered as such.

G/OH was considered to be present if one of the following was observed: 1) if the patient had previously undergone glaucoma surgery in one or both eyes, 2) if the patient was reported to have visual field loss in one or both eyes that was interpreted as glaucomatous, or 3) if the patient was prescribed topical glaucoma treatment during 2015 to 2017. Since data in this study were based on patient records, a distinction between glaucoma and ocular hypertension was uncertain and was therefore not made.

### Visual acuity

In our previous study, visual acuity data were presented only from the seven-year follow-up as more standardised visual acuity testing was undertaken at that time [7]. Data on visual acuity at 24 and 40 years were collected from medical ophthalmic records of routine visits made in different eye clinics all over Sweden. The visual testing at the latter two occasions must therefore be assumed to be of varying precision. To improve comparison of data on visual acuity data from all three follow-ups, the World Health Organization (WHO) classification of visual impairment was used in the present study. This classification divides visual acuity into several groups [14].

### Mortality

In Sweden, registration of the cause of death is mandatory, and as part of the dropout analysis we applied for data on cause of death from the National Cause of Death Register at the National Board for Health and Welfare between 1 January 1992 and 31 December 2017. The individual data obtained were date of death, the main cause of death, medical conditions contributing to the cause of death including diabetes mellitus, if cause of death was investigated through autopsy and if death was declared during hospitalisation. The conditions were coded according to the International Classification of Diseases (ICD), versions 9 and 10.

Through comparison with earlier published data in the field, the cause of death for every patient was interpreted as related to juvenile arthritis or not.

### Statistical analysis

Data were analysed using IBM® SPSS® Statistics (version 27 for Macintosh, IBM Corporation, NY, U.S.A.) For all statistical evaluations, Exact McNemar’s Change Test was used to analyse paired proportions.

## Results

Table 1 presents data on the 25 missing cases. When comparing previously known data on these individuals with data on those participating in the present study no clear differences were seen regarding the distribution of JCA subgroups, presence of anti-nuclear antibodies (ANA) at JCA diagnosis, prevalence of the HLA-B27 haplotype, or the prevalence of severe visual impairment at 7- or 24-year follow-ups.

**Table 1:** Missing patients in the present study mean 40.7-year after uveitis onset

	Deceased before the 40-years follow-up.	Not responding to invitation	Not found	Total
Number of patients	11	10	4	25
Female sex (%)	8	7	3	**18 (72.2)**
EULAR^a^ diagnosis				
O	7	10	4	**21**
P	3	0	0	**3**
S	1	0	0	1
ANA^b^ + (%+)	5	8	4	**17 (68)**
HLA-B27^c^ + (%+)	2	2	1	**5 (20.0)**
Patients with severe visual impairment both eyes^d^				
at the 7.2 -year follow-up	0	1	1	**2**
at 24.0- year follow-up	2	1	0	**3**

^a^ EULAR diagnosis *O* oligoarthritis, *P* polyarthritis, *S* systemic

^b^ Number of patients who were ANA positive at joint disease diagnosis

^c^ Number of patients who had the HLA-B27 haplotype

^d^ According to the World Health Organization classification of visual impairment. Severe visual impairment is defined as a vision score of poorer than 0.1 (Snellen 20/200)

### The present follow-up

The results from the present follow-up are summarised in Table 2.

**Table 2:** Summary of results from the present follow-up at mean 40.7 years after onset of uveitis. The right column shows separate data from the subgroup of 13 patients that were assessed to have active uveitis in this follow-up

	All patients	subgroup of patients with active uveitis
Number of years from onset of uveitis	mean 40.7 ± 6.3 (median 39.0, range 35–63)	39.3 ± 5.2 (median 38.0, range 35–55)
Patient age at follow-up (years)	mean 46.9 ± 6.9 (median 45.5, range 38–72)	46.2 ± 6.2 (median 46.0, range 38–57)
Number of patients participating	30	13
(% of original cohort)	(54.5)	(23.6)
Female sex (%)	16 (53.3)	5
Number of patient records studied	26	13
EULAR^a^		
O	26	12
P	2	0
S	2	1
ANA^b^ at diagnosis (%+)	23+ (76.6)	9+ (69.2)
	6 –	4 -
	1 u	0 u
Female sex with ANA+ (%)	11 (47.8)	3
Presence of HLA-B27^c^ (%+)	8 + (26.6)	5+ (38.4)
	18 –	7-
	4 u	1 u
Female sex with HLA-B27 (%)	2 (25)	1
Patients with cataracts (%)	20 (66.6)	11 (84.6)
Female sex with cataracts	11	5
Patients with glaucom or ocular hypertension (G/OH) (%)	12 (40.0)	8 (61.5)
Female sex with G/OH	5	3
Patients with severe visual impairment both eyes (%)	3 (10)	0
Female sex with severe visual impairment both eyes	1	0
Single eyes with severe visual impairment^d^ (%)	14 (23.3)	5 (19.2)
Female sex, single eyes with severe visual impairment	7	3
Patients with mild or no visual impairment best eye (%)	20 (66.6)	11 (84.6)
Female sex with mild or no visual impairment best eye	11	4
Single eyes with mild or no visual impairment (%)	36 (60)	18 (78.2)
Female sex with mild or no visual impairment	20	6

Severe visual impairment is defined as a vision score of poorer than 0.1 (Snellen 20/200)

Moderate visual impairment is defined as vision equal to or better than 0.1 and poorer than 0.3 (Snellen equal to 20/200 and poorer than 20/70). Mild or no visual impairment is defined as vision equal to or better than 0.3 (Snellen 20/70)

^a^ EULAR diagnosis *O* oligoarthritis, *P* polyarthritis, *S* Systemic

^b^ Number of patients ANA positive (+), negative (−) and unknown (u)

^c^ HLA-B27 positiv (+), negative (−) or unknown (u)

^d^ According to the World Health Organization classification of visual impairment

The four patients that reported not seeing an ophthalmologist regularly in the present study had no ocular complications and good visual acuity in both eyes at the 7-year follow-up, one of the four had active uveitis. At the 24-year follow-up three of these four patients reported not seeing an ophthalmologist regularly and the ophthalmic records of the fourth patient described no signs of active uveitis and full visual acuity in both eyes.

Table 3 shows a comparison between data from all three follow-ups of the studied cohort. Most of the data presented from 7- and 24-year follow-ups have previously been published (Skarin et al. 2009).

**Table 3:** Summary of results from all three follow-up of the same cohort

Mean follow-up time (years) from onset of uveitis	7.2	24.0	40.7
Mean age at follow-up (years)	13.2	31.0	46.9
Female sex (%)	34 (61.8)	29 (59.1)	16 (53.3)
Patients participating (% of original cohort)	55 (100)	49 (89.0)	30 (54.5)
EULAR ^a^			
O	46	41	26
P	6	5	2
S	3	3	2
ANA at diagnosis^b^ (% +)	40+ (72.2)	35+ (71.4)	23 + (76.6)
	13-	12-	6-
	2 u	2 u	1 u
Female sex with ANA+,	24	20	11
HLA-B27^c^ (%+)	13+ (23.6)	13+ (26.5)	8+ (26.6)
	29-	26-	18-
	13 u	10 u	4 u
Female sex with HLA-B27 +,	5	5	2
Patients with active uveitis^d^ (%)	28 (50.9)	27 (55.1)	13 (43.3)
Female sex with active uveitis	19	15	5
Number of patients with cataracts^e^ (%)	23 (41.8)	28 (57.1)	20 (66.6)
Female sex with cataracts	14	19	11
Number of patients with G/OH^f^ (%)	3 (5.4)	12 (24.4)	12 (40.0)
Female sex with G/OH	2	5	5
Number of patients with severe visual impairment in both eyes^g^ (%) of these, female sex	2 (3.6)	5 (10.2)	3 (10.0)
	2	3	1
Number of single eyes with severe visual impairment ^g^ (%) of these, female sex	12 (10.9)	17 (17.3)	14 (23.3)
	8	11	7
Number of patients with mild or no visual impairment in best eye (%) of these, female sex	49 (89.0)	29 (59.1)	20 (66.6)
	30	19	11
Number of single eyes with mild or no visual impairment (%) of these, female sex	84 (76.3)	49 (50.0)	36 (60.0)
	51	30	20

Severe visual impairment is defined as a vision score of poorer than 0.1 (Snellen 20/200)

Moderate visual impairment is defined as vision equal to or better than 0.1 and poorer than 0.3 (Snellen equal to or better than 20/200 and poorer than 20/70)

Mild or no visual impairment is defined as vision equal to or better than 0.3 (Snellen 20/70)

^a^ EULAR diagnostic classification at onset of Juvenile Chronic Arthritis: O=oligoarthritis, P=polyarthritis, S=systemic disease

^b^ Number of patients who were ANA positive (+), negative (-) or unknown (u) at diagnosis of joint disease

^c^ HLA-B27 positive (+), negative (-) or unknown (u)

^d^ The difference in the incidences of active uveitis at 7, 24, and 40 years was not significant

^e^ The increase in the number of patients with cataracts between 7 years and 24 years was significant (*p*=0.001), while the difference between 24 years and 40 years was not (*p*=1.000)

^f^ The increase in the number of patients with G/OH was statistically significant between the 7-year follow-up and the 24-year follow-up (*p*=0.004), while the difference between 24 and 40 years was not (*p*=0.250)

^g^ According to the World Health Organization classification of visual impairment

### Active uveitis

In the present follow-up uveitis was reported to be active in 43.3% (13/30) which corresponds to 23.6% (13/55) of the whole original cohort (Tables 2 and 3).

A comparison between all participants in the present study and the subgroup with active uveitis is presented in Table 2. Prevalence of cataracts and glaucoma was slightly higher in the subgroup with active uveitis at the time of the present study, but neither was significantly different from that of the whole group. No cases of severe visual impairment in both eyes were seen in the subgroup with active uveitis.

### Cataracts and cataract surgery

In the 40-year follow-up study, 20/30 (66.6%) of the participants had cataracts or had undergone cataract surgery in one or both eyes (Tables 2 and 3). When adding all reported cases with cataracts, a total of 34/55 (61.8%) patients in the original cohort were reported to have cataracts or had undergone cataract surgery at any of the three follow-ups. No new cases of cataracts were reported between 24-year and 40-year follow-ups. The postoperative status following cataract surgery was not consistently reported. Of 21 eyes for which the postoperative status was clearly described, 10 were aphakic and 11 were pseudophakic. Of these 21 eyes, six had severe visual impairment; two of which were aphakic and four pseudophakic. Of the remaining 15 eyes, 12 had mild or no visual impairment; six of which were aphakic and six pseudophakic. Thus, no clear difference was seen in postoperative visual acuity between patients who had undergone lensectomy compared with standard cataract surgery with implantation of an intraocular lens.

### G/OH

The number of patients with G/OH are presented in Tables 2 and 3. Four of the individuals with G/OH at 24 years’ follow-up had died before the present study commenced and four additional patients were reported to have G/OH at 40-year follow-up. In total, 16/55 (29.0%) in the whole original cohort were reported to have G/OH at any of the three follow-ups.

### Visual impairment

In our previous study only visual acuity data from the 7-year follow-up were presented [7]. In the present study the numbers of single eyes and individual patients who were reported to have severe visual impairment or worse using the WHO classification scale and the numbers of patients with mild or no visual impairment at 7, 24, and 40 years are presented in Tables 2 and 3.

When adding results from the previous two follow-ups, 12.7% (7/55) of the original cohort were reported to have severe visual impairment in both eyes at any of the three follow-ups.

### Mortality

A total of 11 patients had died before the present study. Two of these had died before the 24-year follow up [7]. The median age at death was 48 years and 8/11 (72.7%) of the deceased were female. Seven (63.6%) were subcategorized as having oligoarticular JCA, three (27.3%) as polyarticular JCA and one individual (9.1%) had systemic JCA according to the EULAR criteria.

### Causes of death

Rheumatic disease was stated as the main cause of death in four of the cases. Juvenile arthritis was recorded as a contributory condition for an additional three cases. When weighing all contributory conditions together for every patient, only one death was interpreted as being caused by a direct complication of juvenile arthritis. Malignancy and cardiovascular disease were not considered to be complications of juvenile arthritis.

## Discussion

The aim of the present study was to continue following the same uveitis cohort into midlife regarding active uveitis, ocular complications, and visual impairment [7]. The retrospective design based on review of patient medical records limits the amount of data that could be evaluated.

Lack of information on 25 patients makes our findings less reliable than those of our previous follow-up studies of the same cohort. As 11 of these were deceased it should be noted that the participating 30 patients represent 68% of those 44 individuals who were still alive at the time of the study.

Only four of the 30 patients participating in the current study did not attend regular ophthalmology visits. It is therefore reasonable to assume that the majority of those willing to participate were individuals with concerns about their eyes or eyesight because of ocular symptoms, and therefore might not constitute a random sample of all the 44 patients living at the time of the current study.

In our 24-year follow-up, 34/55 patients (61.8%) reported seeing a rheumatologist regularly [7]. It is thus possible that the rheumatological diagnosis of some of these patients might have been revised during these 40 years, as has been described previously [15].

The prevalence of ANA was significantly higher in our original cohort (72.7%) than in children with chronic uveitis described in a Finnish study and a more recent Nordic JIA study, which was 37 and 47.4% respectively [5, 6].

In the 1970s and 1980s in Sweden, a different immunofluorescence substrate (rodent liver cells) was used as a substrate as opposed to cultured HEP-2 cells used in a more recent Nordic study [6, 7]. This change in test methodology might have contributed to a change in sensitivity. Other studies describing JCA and JIA in patients diagnosed in the 1970s and 1980s reported a prevalence of ANA in patients with uveitis similar to that in our cohort. None of these studies reported which method was used for the detection of ANA [12, 13].

Previous long-term follow-up studies have presented data on the second and third decade after onset of joint disease or uveitis. The data presented in these studies regarding signs of active uveitis, cataracts, and glaucoma/ocular hypertension accord well with those of our cohort at the 24-year follow-up [5, 7, 10, 11, 13]. The results of more recent studies from several countries indicate a lower prevalence of cataracts during the initial years after onset of uveitis than in our older Swedish cohort. The reduced prevalence of cataracts in more recent reports might be ascribed to newer treatment regimens which might more efficiently suppress uveitis [16].

It is likely that the earliest cases of G/OH in our original cohort of 55 patients (5.4% at seven years) were mainly patients who developed increased intraocular pressure due to topical steroid treatment, known as ‘steroid responders’. Increased intraocular pressure due to topical steroids typically appears within weeks to months after the start of treatment. The cases of G/OH appearing after the 7-year follow-up were more likely to be the type observed in many patients with long-standing chronic uveitis, where a gradual increase in intraocular pressure is assumed to be caused by impaired aqueous outflow due to increasing tissue damage in the aqueous outflow system caused by the inflammatory process [17].

The fact that 13% of our original cohort were reported to have severe visual impairment or worse in both eyes at any of the three follow-ups is noteworthy. In a recent study of a German population concerning visual impairment, with individuals of approximately the same age as in the present follow-up, the corresponding number was below 0.5% [18].

In previous long-term studies of young adults with JIA-associated uveitis where visual acuity was reported, the prevalence of severe visual impairment according to the WHO classification ranged from 2 to 8% of single eyes in Danish and Finnish studies [5, 13], respectively, to 12% of single eyes in a Canadian study [10]. The Canadian data accord relatively well with those from the present cohort at all three follow-ups, while the numbers from the other two studies are lower.

A majority of the patients in our original cohort were female. It is noteworthy that, although the numbers are smaller in this 40-year follow-up, the sex distribution among those with active uveitis after 40 years is reversed. A trend towards reducing the sex difference was also observed with respect to the prevalence of severe visual impairment. These findings support data from studies with a shorter follow-up time reporting that male sex can be associated with a worse clinical outcome [19].

The patients in our cohort were initially treated according to best practice treatments that were available in the late 1970s and early 1980s. Modern JIA treatments with DMARDs appear to be more successful in minimising the acute and severe consequences of both joint disease and uveitis, but do not appear to reduce the incidence of uveitis significantly in patients with this diagnosis [2, 6, 20, 21].

Prospective studies of adult JIA patients are needed to clarify better how both systemic and topical eye treatments should be optimised to reduce the risk of visual impairment.

The mortality rate of 20% was surprisingly high in this cohort. For comparison, the corresponding figure for all individuals in the Skåne Region of Sweden born 1960–1979 was 2.46% in the year 2020 (Jacobsson H, personal communication).

In studies of patients with juvenile arthritis with shorter follow-up times, no increased mortality rate was reported compared with the general population [22, 23]. These findings accord well with respect to follow-up time and results of our seven-year follow-up where no deaths were reported. However, in a population-based study from Minnesota with a mean follow-up of 25.6 years, four deaths occurred of which all were linked to complications of comorbid autoimmune conditions [24]. Other studies have found increased mortality among patients with juvenile arthritis compared with groups of patients with other rheumatic diseases [24–26]. The participants in our cohort were diagnosed 1945–1982, which is earlier than the above-mentioned studies, at a time when the pharmacological treatment options for rheumatic diseases were limited and not as effective as the immunomodulatory medicines today. This might be a fact contributing to the high mortality rate in our present follow-up.

Only data from ophthalmic records were collected in this study. Thus, we do not have information on systemic inflammatory activity and the systemic pharmacological treatment prescribed during the disease course, which prevents us from hypothesising about possible connections between the inflammatory disease and the causes of death.

There are studies showing an increased risk of malignancy in JIA [27–29], but whether that is a result of the disease itself or the pharmacological treatment given is not determined. Other studies have shown the opposite results [30, 31].

JIA increases the risk of serious bacterial infections [27], and treatment with disease modifying anti-rheumatic drugs (DMARDs) seems to increase that risk slightly more [27, 32].

Our findings accord with data that describe increased mortality in adult patients with juvenile arthritis. However, our cohort is small and limited to the subgroup of JIA patients with uveitis treated at a tertiary centre, which may indicate more severe disease.

## Conclusion

We present unique data from a third follow-up of a patient cohort diagnosed with uveitis associated with juvenile arthritis for a mean 40.7 years after the uveitis onset. Our data support previous findings indicative of a possible higher mortality rate in adult patients with juvenile arthritis than in the rest of the population.

Four decades after diagnosed with JCA-associated uveitis, approximately one-quarter of the patients were reported to have active uveitis. Our data indicate that the total number of patients that were reported to have cataracts, G/OH and severe visual impairment in both eyes increased throughout our three follow-ups. Our findings support the continuation of regular ophthalmic check-ups throughout life in patients diagnosed with JCA- and JIA-associated uveitis.

## Data Availability

The datasets used and/or analysed during the current study are available from the corresponding author upon reasonable request.

## Abbreviations

JIA: Juvenile Idiopathic Arthritis
JCA: Juvenile Chronic Arthritis
EULAR: The European League Against Rheumatism
G/OH: Glaucoma or ocular hypertension
WHO: World Health Organization
ICD: International Classification of Diseases
ANA: Anti-nuclear antibodies
HLA-B27: Humen Leucocyte Antigen B27
DMARD: Disease Modifying Anti-Rheumatic Drugs
